# Expectancy after the first treatment and response to acupuncture for menopausal hot flashes

**DOI:** 10.1371/journal.pone.0186966

**Published:** 2017-10-27

**Authors:** Carolyn C. Ee, Sharmala Thuraisingam, Marie V. Pirotta, Simon D. French, Charlie C. Xue, Helena J. Teede

**Affiliations:** 1 National Institute of Complementary Medicine, Western Sydney University, Sydney, New South Wales, Australia; 2 Department of General Practice, University of Melbourne, Melbourne, Victoria, Australia; 3 School of Rehabilitation Therapy, Queens University, Kingston, Ontario, Canada; 4 Department of Chiropractic, Faculty of Science and Engineering, Macquarie University, New South Wales, Australia; 5 School of Health and Biomedical Sciences, RMIT University, Melbourne, Victoria, Australia; 6 Monash Centre for Health Research and Implementation: a partnership between Monash Health and the School of Public Health, Monash University, Melbourne, Victoria, Australia; Weill Cornell Medical College Qatar, QATAR

## Abstract

**Background:**

Evidence on the impact of expectancy on acupuncture treatment response is conflicting.

**Objectives:**

This secondary analysis of a randomized sham-controlled trial on acupuncture for menopausal hot flashes investigated whether treatment expectancy score was associated with hot flash score at end-of-treatment. Secondary analyses investigated whether there were associations between other pre-specified factors and hot flash score.

**Study design:**

Women experiencing moderately-severe hot flashes were randomized to receive 10 sessions of real or sham acupuncture over eight weeks. Hot flash score was collected using a seven-day hot flash diary, and expectancy using the modified Credibility and Expectancy Questionnaire immediately after the first treatment. Linear mixed-effects models with random intercepts were used to identify associations between expectancy score and hot flash score at end-of-treatment. Regression was also used to identify associations between pre-specified factors of interest and hot flash score. Because there was no difference between real and sham acupuncture for the primary outcome of hot flash score, both arms were combined in the analysis.

**Results:**

285 women returned the Credibility and Expectancy Questionnaire, and 283 women completed both expectancy measures. We found no evidence for an association between expectancy and hot flash score at end-of-treatment for individual cases in either acupuncture or sham group. Hot flash scores at end-of-treatment were 8.1 (95%CI, 3.0 to 13.2; P = 0.002) points lower in regular smokers compared to those who had never smoked, equivalent to four fewer moderate hot flashes a day.

**Conclusion:**

In our study of acupuncture for menopausal hot flashes, higher expectancy after the first treatment did not predict better treatment outcomes. Future research may focus on other determinants of outcomes in acupuncture such as therapist attention. The relationship between smoking and hot flashes is poorly understood and needs further exploration.

## Introduction

Acupuncture is a Chinese medical intervention that involves the insertion of fine metal needles into specified areas of the body. Several large meta-analyses of acupuncture for painful conditions have demonstrated that acupuncture produces small specific effects and considerable non-specific effects [1–3]. Critics of acupuncture assert that its clinical response is by and large due to high expectations of the patients[4]. However, the empirical evidence for or against the influence of expectancy on acupuncture treatment outcome is contradictory.

Expectancy, or its synonym expectations, refers to an individual’s beliefs about future events[5]. Treatment expectancy can have a positive or negative impact on treatment outcome and is a major contributor to the placebo response[6]. Expectations may vary according to individual understanding about the illness, treatment, and experiences with past treatments, and may change over the course of treatment [5].

Two systematic reviews of acupuncture trials that measured the impact of participants’ expectations on outcomes have reported mixed findings. Colagiuri and Smith reviewed findings from nine acupuncture trials[7], of which the majority examined acupuncture for painful conditions and used mainly self-reported outcomes. Four trials failed to find any effects of expectancy on outcomes, while five found at least some evidence that higher expectancy predicted better outcomes. Prady et al reviewed 58 trials, similarly most on painful conditions, and again found conflicting evidence[8]. In more recent research, Vase and colleagues demonstrated that perceived treatment allocation (to real or sham acupuncture) resulted in significantly lower dental pain levels[9], and Bauml and colleagues reported that higher expectancy predicted higher treatment response for sham but not for real electro-acupuncture in a trial of acupuncture for joint pain in breast cancer patients[10]. There is limited evidence on the impact of expectancy on acupuncture treatment outcomes for conditions other than pain.

Hot flashes (HFs) cause significant morbidity in menopausal women[11, 12] and last an average of five years[13]. Because of perceived shortcomings of conventional medical treatment, complementary therapies for hot flashes have increased in popularity[14] and are used by up to 80% of women during the menopause[15]. Acupuncture is a popular treatment amongst menopausal women[16] however there is insufficient evidence of its efficacy as a treatment for hot flashes[17]. We conducted a randomized sham-controlled trial testing the efficacy of acupuncture for hot flashes[18]. As both real and sham acupuncture groups improved equally in our study, we now aim to examine the impact of treatment expectancy on the acupuncture response. The primary aim of this post hoc analysis was to investigate whether treatment expectancy score (measured at the end of the first acupuncture treatment) was associated with HF score at end-of-treatment.The secondary aims were to determine other factors associated with hot flash score at end-of-treatment.

## Materials and methods

This is an *a priori* planned secondary analysis of data from a randomized controlled trial (RCT) that investigated the effects of acupuncture compared to sham on menopausal hot flashes. The protocol[19] and key findings[18] from the RCT have been published. Ethics approval was obtained from the Human Research Ethics Committee, University of Melbourne and the trial was prospectively registered on the Australian and New Zealand Clinical Trial Registry http://www.anzctr.org.au (ACTRN12611000393954).

### Participants

We recruited 327 women having at least seven moderately-severe HFs a day from the community, in metropolitan and regional areas of Australia. Participants were aged over 40 years old and were postmenopausal or in the late menopausal transition. Exclusion criteria included current use of menopausal hormone therapy, other HF treatments within the preceding 12 weeks, bilateral salpingo-oophorectomy, breast cancer, and needle acupuncture within the last two years.

### Intervention and control

Women were randomized to receive real acupuncture (based on Chinese Medicine principles) or non-invasive sham acupuncture. Ten treatments were given over eight weeks, twice weekly for the first fortnight and weekly thereafter. Participants were blinded to treatment allocation, but acupuncturists were not.

### Outcomes

The trial primary outcome was change in HF score at end-of-treatment. HF frequency, severity and a composite HF score were calculated from validated seven-day HF diaries[20] collected at baseline, four weeks, end-of-treatment (eight weeks), and three- and six-month after end of treatment. At baseline, we collected information on known risk factors for HFs. From these, we selected variables that we hypothesised were most likely to influence hot flash score at end-of-treatment. We did not include variables with a significant proportion of missing data (such as income) or little variation between categories (such as ethnicity). The chosen variables are summarised in Table 1.

**Table 1:** Information about potential confounders, collected at baseline.

Potential confounder	Categories
**Current smoking status[21]**	Never smoked, used to smoke, now smoke occasionally, or now smoke regularly
**Education level[22–24]**	Highest level of education attained (primary school, high school, college/vocational training/university degree, or postgraduate);
**Duration of hot flashes in years**	
**Anxiety score[25]**	Anxiety subscore from Hospital Anxiety and Depression Scale
**Perceived treatment allocation**	Real/placebo/not sure
**Practitioner gender[26]**	Male/female practitioner
**Credibility score[27]**	9 point Likert scale: 1 = “not at all useful”; 9 = “very useful”

### Expectancy

We used a modified version of the Credibility and Expectancy Questionnaire (CEQ) to measure expectancy. The CEQ is a validated, widely-used, quick and easy-to-administer 6-item scale for measuring treatment expectancy and rationale credibility in clinical outcome studies[27]. We administered this questionnaire immediately after the first treatment in order to capture expectancy levels following the experience of a treatment but before expectations are influenced by clinical improvement. Treating acupuncturists gave the participant a questionnaire to complete at the end of the first treatment and then returned the questionnaires to us by reply-paid mail.

The CEQ consists of two sections: the first asks participants what they think will happen at the end of treatment, and the second asks what they feel will happen. This distinguishes between cognitive and affective aspects of belief, which can be distinct; one can hold rational thoughts which conflict with a sense of hope or faith[27]. The first section contains four questions, three on credibility (logicalness of the treatment, success in reducing symptoms, confidence in recommending to a friend) and one expectancy question (improvement in symptoms by the end of treatment). The second section asks one credibility question (success of treatment) and one expectancy question (improvement in symptoms) but distinguishes these as how the patient feels about improvement rather than thinks. The original CEQ used a Likert scale from 1–9, 1 representing “not at all” and 9 representing “very much” or “very useful” for the credibility questions, and a scale from 0–100% in 10% increments for the expectancy questions. The midpoint was described as “somewhat useful”. To simplify the questionnaire we applied the same 9-point Likert scale to the expectancy questions. A composite score was derived for the four credibility questions, and for this analysis we calcuated mean scores for each of the two expectancy questions.

As surveys to measure the success of blinding may enhance participants focus on this, we measured this aspect immediately after the first treatment[28], by inserting a question at the end of the CEQ which asked women to indicate which treatment they believed they had received, with answer options of “real acupuncture”, “sham acupuncture” and “not sure”.

As all outcomes were self-assessed, outcome assessment was considered blinded as well.

### Analysis

Analysis was restricted to the 285 women who provided CEQ data. The two expectancy items (“Expectancy 1 and 2”) were included in the analysis and the wording of these questions is presented in Table 2.

**Table 2:** Wording of expectancy questions in the Credibility Expectancy Questionnaire.

Question	Range
**“Expectancy 1”: By the end of the treatment period, how much improvement in your hot flashes do you think will occur?**	1 = None at all
	9 = Total improvement
**“Expectancy 2”: By the end of the treatment period, how much improvement in your hot flashes do you really *feel* will occur?**	1 = Not at all
	9 = Very much

Stata version 13.1 (StataCorp) was used for all analyses. Since the previous study did not find a difference in outcome between the treatment groups, study arm was not included as a confounder in this analysis. Instead, data for both arms were combined and analysed as one. Bivariate analyses were performed to identify associations between independent variables and hot flash score at end of treatment with adjustment for baseline hot flash score. The independent variables tested were expectancy, HF duration (years), baseline anxiety, practitioner gender, perceived treatment allocation, smoking status, credibility and education. The final model consisted of all variables found to be statistically significant in the bivariate analyses and the multivariable analyses at the 0.05 alpha significance level. Both a log and square root transformation of the outcome variable were considered to minimise heteroscedasticity.

All models in the analyses were linear mixed-effects models with random intercepts. Restricted maximum likelihood estimation (REML) was used to estimate the effect of expectancy score and the other independent variables on hot flash score. These estimates, with 95% confidence intervals and p-values, were reported. Missing data was assumed to be missing at random in these analyses.

The goodness of fit of the models was assessed using residual plots (results not shown) and the impact of potential outliers on regression results explored. No imputation methods were carried out, however, a pattern-mixture model was used to evaluate the impact of the assumption of missing at random (see S1 Fig).

## Results

**Fig 1:**
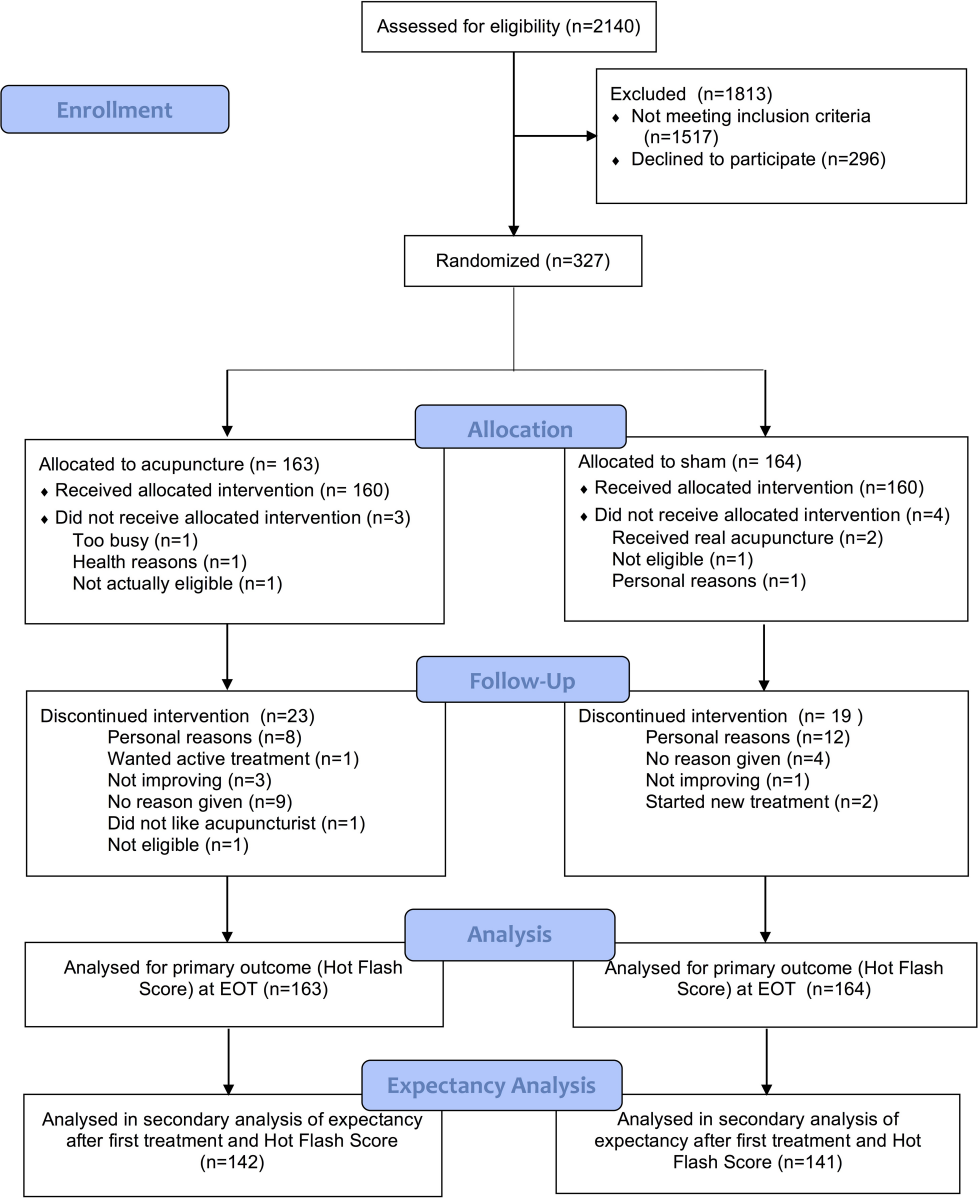
Flowchart of trial procedures.

285 women returned the CEQ, and 283 completed both expectancy questions. Table 3 describes the demographic details of the participants. Participants were mainly Caucasian, well educated and had good to excellent self-rated health levels. The mean age was 55 years. No serious adverse events were reported. Fig 1 depicts the flow of participants in the study through to end of treatment (eight weeks).

**Table 3:** Participant baseline demographic characteristics.

N = 285		Missing
Characteristic	n (%)	n (%)
**Age (years)**	54.9 (4.1)*	37 (13.0)
**Ethnicity**		39 (13.7)
** Caucasian**	237 (96.3)	
** Non-Caucasian**	9 (3.7)	
**Highest educational attainment**		38 (13.3)
** Primary or high school**	93 (37.7)	
** Vocational training, diploma, university degree or higher**	154 (62.4)	
**Average weekly household income**		89 (31.2)
** Low income ($0–599)**	76 (38.8)	
** Middle income ($600–2000)**	111 (56.6)	
** High income (≥$2000)**	9 (4.6)	
**Self-rated health**		39 (13.7)
** Excellent**	47 (19.1)	
** Very good**	111 (45.1)	
** Good**	73 (29.7)	
** Fair**	11 (4.5)	
** Poor**	4 (1.6)	

We found no evidence of an association between expectancy after the first treatment and HF score at the end of the treatment period. On average, HF scores at end of treatment were 8.1 (95%CI, 3.0 to 13.2; P = 0.002) points lower in regular smokers compared to those who had never smoked. There was a lack of evidence to suggest an association between the other independent variables and HF score at end of treatment. Table 4 summarises the findings from the bivariate analyses of the independent variables and HF score.

**Table 4:** Bivariate associations between independent variables and hot flash score at EOT adjusted by baseline hot flash score (N = 285).

Independent Variables	n*	Missing n (%)	Mean change in hot flash score at EOT*	*P*-value
**Expectancy question 1 (1–9)**	6.8 (1.6)**	1 (0.4)	-0.3 (-1.1 to 0.4)	0.42
**Expectancy question 2 (1–9)**	6.9 (1.7)**	2 (0.7)	-0.3 (-1.0 to 0.4)	0.35
**Credibility score (composite) (1–9)**	6.8 (1.5)**	-	0.02 (-0.8 to 0.8)	0.97
**Hot flash duration (years)**	5.1 (4.6)**	40 (14.0)	0.1 (-0.16 to 0.38)	0.42
**Anxiety score**	7.6 (4.2)**	35 (12.3)	-0.2 (-0.5 to 0.1)	0.30
**Practitioner gender**		9 (3.2)		0.96
** Female practitioners**	98		-	
** Male practitioners compared to female practitioners**	178		0.03 (-1.3 to 1.4)	
**Perceived study arm allocation**		5 (1.8)		0.89
** “Sham acupuncture”**	13		-	
** “Real acupuncture” compared to “sham acupuncture”**	91		-1.3 (-6.7 to 4.1)	
** “Not sure” compared to “sham acupuncture”**	176		-1.0 (-6.3 to 4.3)	
**Smoking status**		37 (13.0)		0.02
** Never smoker**	116		-	
** Regular smoker compared to never smoker**	21		-8.1 (-13.2 to -3.0)	
** Occasional smoker compared to never smoker**	8		-0.9 (-7.7 to 5.8)	
** Previously smoked compared to never smoked**	103		-0.7 (-3.3 to 2.0)	
**Education**		38 (13.3)		0.61
** Completed primary school or high school**	93		-	
** Completed a degree compared with primary school/high school**	109		-1.4 (-4.3 to 1.4)	
** Completed postgraduate education compared with primary school/high school**	45		-1.00 (-4.6 to 2.6)	

*number of participants with a HF score and response for the independent variable listed. There were 37 (13%) participants with missing HF score at end of treatment.

**Mean (SD)

EOT = end of treatment

There were a few participants with large HF scores. These were found to have little influence on the regression model. A log or square root transformation of the outcome variable did not seem to significantly reduce heteroscedasticity or change study conclusions.

The sensitivity analysis for missing data (S1 Fig) showed that mean hot flash scores would be no different between regular and never smokers if regular smokers with missing data had a mean hot flash score 10 points greater than the observed regular smokers. This difference is larger than the standard deviation of hot flash scores for regular smokers and seems implausible.

## Discussion

In this post hoc analysis of a randomized sham-controlled trial of acupuncture for menopausal hot flashes, we did not find evidence that higher expectations, perceived treatment allocation or credibility score after the first treatment predicted a better treatment response to acupuncture. Each one-point increase in expectancy score resulted in an estimated reduction of one third of a mild hot flash per day, which is not clinically significant.

In previous trials of acupuncture for hot flashes, there is limited and inconclusive evidence on the impact of expectancy on outcomes. In one large pragmatic trial comparing acupuncture with usual care, positive expectations were not associated with treatment outcome[29]. In contrast, high expectancy predicted a greater response to treatment in the acupuncture group in another open-labelled trial[30]. Meanwhile, participants in the real acupuncture group in a sham-controlled pilot study had higher expectations than in the sham group but it is unclear if this resulted in better outcomes[31]. Taken together, there is little evidence for an association between expectancy and improvement in HFs in acupuncture trials, particularly in Western countries.

The only variable that predicted treatment outcome in our study was smoking status. Being a current smoker was associated with a clinically significant difference equivalent to four fewer moderate hot flashes a day, when comparing never smokers with regular smokers, and is consistent with a recent finding of the association between smoking status and a higher placebo response in a trial of a pharmaceutical for hot flashes[21]. However, this was not mediated by expectancy levels in our study. The reasons for this association are unclear. Smoking is a risk factor for experiencing HFs[24, 32] and smokers tend to over-report the frequency of their hot flashes[33], rate their hot flashes as more problematic[34] and report higher levels of bother from their hot flashes than non-smokers with a similar HF burden[35]. This finding should be interpreted with caution as the proportion of smokers in our study was small. We also did not collect data on smoking cessation during the trial, and do not know if these women’s symptoms improved because they stopped smoking.

Henry Beecher reported that about 30% of patients responded positively to a placebo treatment. However, in doing so, he failed to distinguish the placebo response from other confounding factors, therefore over-inflating the perceived power of the placebo[6]. This error has persisted, and today the placebo response is frequently confused with the overall improvement that occurs in a placebo control group, which is a composite of the psychobiological placebo response and other reasons for clinical improvement such as regression to the mean.

The placebo response is a distinct psychobiological response seen when a placebo is administered, mediated by opioidergic and dopaminergic pathways and modified by psychosocial context[36]. This response can be reversed by the opioid antagonist naloxone and can also have non-opioidergic effects[6]. It is not equivalent to the overall response seen in control groups, and should be carefully distinguished from other factors that can induce a clinical response. Expectancy and classical conditioning are the principal mechanisms of the placebo response, however in this study we failed to find an association between expectancy and treatment outcome. This suggests that the improvement in HFs is mediated by factors other than the physiological placebo response.

Non-placebo improvements can be seen in active, control and placebo groups. Here, improvements in both groups may have been due to response bias. Research participants can become attached to the research team and committed to success of the trial, which may predispose them to report improvements in symptoms[37]. Alternatively, participants may have felt compelled to rate their expectations higher than they actually were.

Another likely explanation for clinical improvement in our study may be the frequent therapist interaction that is inseparable from the act of receiving acupuncture needling. Although earlier studies suggested that empathic consultation styles predicted treatment outcomes in acupuncture consultations[38, 39], more recent studies have not replicated this finding[26]. However, qualitative research by Paterson and colleagues indicate that patients attending for acupuncture consultations value the “*talking*, *listening and practitioners’ attention to them as an individual*”[40] and practitioner characteristics of being “*considerate*, *respectful*, *caring*, *kind*, *understanding*, *professional*, *helpful*, *reassuring and supportive*”. Also, patients in Paterson’s study valued the interactions with clinic staff and the friendly atmosphere of acupuncture clinics. In particular, practitioner empathy has been linked to patient enablement[38], which suggests that participants in our study may have developed new ways to cope with their symptoms and new self-care strategies, as a result of interacting with an empathic practitioner. This is supported by findings from antidepressant trials reporting markedly improved outcomes when participants had a greater number of treatment visits[41] and findings from a Cochrane review on placebo interventions which found larger clinical improvements with physical placebos compared with placebo pills, particularly with those that required the placebos to be administered by a therapist[42, 43]. In a mixed-methods study on acupuncture for dysmenorrhoea, participants described a feeling of partnership with their practitioners, validation of their symptoms, and a sense of hope arising from the acupuncture consultations, which contributed to their overall improvement[44]. Future research may need to study non-needling effects of acupuncture including assessment of the quality of the therapeutic relationship, and level of practitioner empathy.

Another likely reason for clinical improvement is the physiological effect of sham needling. Sham acupuncture blinds participants and controls for both expectations from receiving acupuncture and for other effects not associated with needling, including seeing a therapist. This minimises performance and detection bias, particularly if outcomes are self-reported[43]. However, light stimulation of the skin can activate descending pain modulation pathways, particularly with repeated stimulation[45]. Sham acupuncture is widely recognised as a “non-inert” comparator, and has been criticised as an inadequate control method for acupuncture[46, 47]. The effectiveness of “placebo devices” for acupuncture has been comprehensively reviewed recently[48] and although the Park Sham Device used in our study appears to be superior to other non-invasive devices, none of the currently available sham control methods are considered inert and certainly none are equivalent to placebo.

Strengths of the main acupuncture study include a broad recruitment strategy, successful blinding of participants, high retention rate, and approximately equal proportions of participants with previous acupuncture experience and no previous acupuncture experience. Additionally, although this was a secondary analysis, it was considered *a priori*[19] therefore adding to the rigour of our analysis.

Some limitations must be considered when interpreting our findings. First, our primary aim was to assess the efficacy of acupuncture for hot flashes, and not to evaluate the impact of expectancy on treatment outcome. Second, we only measured expectations immediately after the first treatment and it is possible that expectations change over time especially in response to a perceived improvement in symptoms. Third, 13% of women did not return surveys on expectancy, therefore the impact of expectations in these women is unknown; as well as this, we had a number of missing responses to the other variables. Also, although we used a validated and widely-used expectancy scale, treatment expectancy is difficult to measure. For participants to respond accurately they must interpret the question correctly, be aware of their own expectations and understand the rating scale of the question. Also, we modified the CEQ by using the 9-point Likert scale instead of a scale from 0–100% for the expectancy questions, although we think the impact on our findings would be minimal. Fourth, we did not include a no-treatment control group, and do not have data on the true inert placebo effect in our study, that is, given sham acupuncture appears not to be equal to placebo.

Finally, expectancy is but one of the psychological mechanisms contributing to the placebo response, albeit a key mechanism. Other mechanisms contributing to the placebo response include classical conditioning, learning, memory, motivation, somatic focus, anxiety reduction and meaning[6] and it is possible that some of these may have played a role in treatment outcome. In a qualitative study of participants of a sham-controlled trial of acupuncture for irritable bowel syndrome, Kaptchuk and colleagues found that the experiences of participants were complex and idiosyncratic, concluding that the placebo response in a randomized placebo-controlled trial context is not a simple and linear construct[49].

## Conclusions

Findings from this post hoc analysis of our large randomized clinical trial of acupuncture for menopausal hot flashes demonstrate that there was no evidence for an association between expectancy measured immediately after the first treatment, and treatment outcome. Our findings refute the assertion that the effects of acupuncture are driven by patient expectations. Rather, the non-specific effects of acupuncture may relate more to other factors that can influence response to comparators or placebos in clinical research including therapist attention and increased sense of enablement.

The role of sham controls in acupuncture trials should be revisited and suitable comparators for acupuncture should be considered in future research, such as comparing acupuncture to equivalent attention interventions. We also found that being a regular smoker was associated with a large clinical improvement; the reasons for this are unclear but should be explored further.

## Supporting information

S1 Fig Sensitivity analysis. (TIFF)

S1 File Dataset. (XLS)
