# Supplementary figures and images for: Expectancy after the first treatment and response to acupuncture for menopausal hot flashes
Source: PLoS One. 2017 Oct 27;12(10):e0186966. doi: 10.1371/journal.pone.0186966 (PMC5659680; doi:10.1371/journal.pone.0186966)

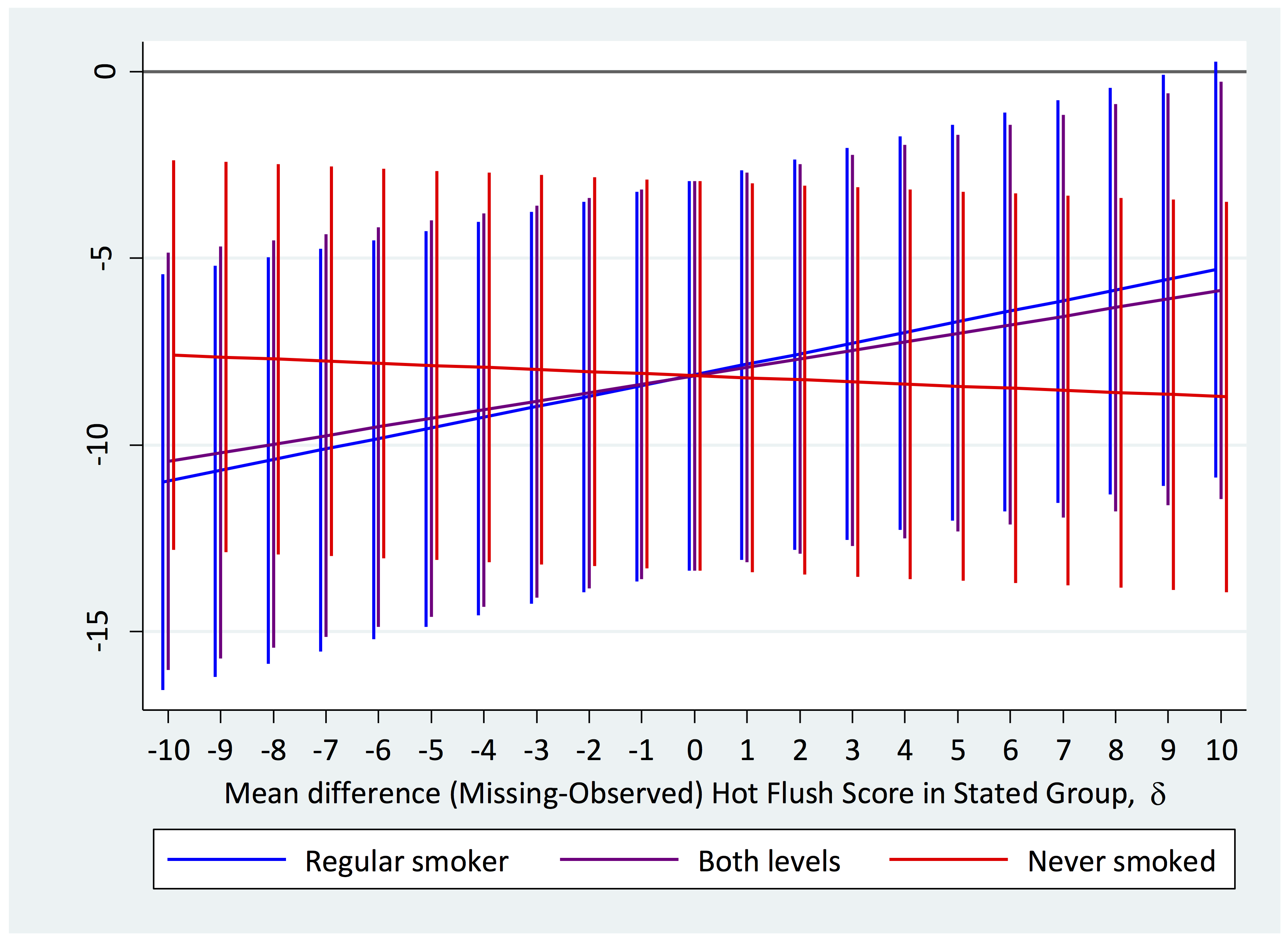

Supplement: S1 Fig — (TIFF) [file pone.0186966.s001.tiff]
